# Transient neonatal zinc deficiency in a 3-month-old infant: A case report

**DOI:** 10.1177/2050313X251341516

**Published:** 2025-05-13

**Authors:** Damy Horth, Isabelle Auger

**Affiliations:** 1 Department of Dermatology, Université Laval, Québec, QC, Canada

**Keywords:** pediatric dermatology, dermatology, zinc deficiency, transient neonatal zinc deficiency, TNZD

## Abstract

Zinc deficiency can be inherited, such as in the case of acrodermatitis enteropathica or acquired. In both cases, patients will present typical acral and periorificial skin lesions, and seldom, diarrhea as well as secondary alopecia. In this report, we provide a case of transient neonatal zinc deficiency in a 3-month-old breastfed girl who presented with classical skin lesions of zinc deficiency. The diagnosis was determined following the results of blood tests, which indicated a combination of low plasma phosphatase alkaline and zinc levels. To support the diagnosis, genetic testing was performed on the mother to detect a mutation in the SLC30A2 gene. However, the results were inconclusive as a variant of unknown significance was found. After starting zinc supplementation, the skin lesions completely resolved a few weeks later without any recurrence.

## Introduction

Transient neonatal zinc deficiency (TNZD) is an acquired form of zinc deficiency secondary to a decreased excretion of zinc in breast milk. The deficiency is caused by a mutation carried by the mother in the SLC30A2 gene encoding zinc transporter 2 (ZnT2), which is a zinc carrier protein in the glandular epithelial mammary cells.^1^ Based on previous observation, the gene mutation has been associated with a reduction of up to 75% of normal zinc levels in breast milk.^2^ The condition, occurring exclusively in breastfed newborns, tends to resolve when complementary feeding with solid food is instituted,^3^ although the consequences can be severe if no treatment is started beforehand.

It contrasts with the classic presentation of acrodermatitis enteropathica (AE), which is a rare autosomal recessive condition secondary to SLC39A4 gene mutation carried by the infant encoding zinc/iron-regulated transporter-like protein (hZIP4), a ZnT within the intestinal epithelium.^4^ This inherited condition tends to occur in newborns who are either fed through formula or once they are weaned off from breastfeeding. Indeed, given that normal breast milk carries a high concentration of zinc and contains ligands that increase the bioavailability of zinc,^5^ symptoms of AE will become apparent once an infant with AE no longer consumes breast milk.

The diagnosis of TNZD is made with classical clinical manifestations and by a reduced plasmatic zinc level among other plasmatic markers. A decreased level of zinc in the breast milk or genetic testing in the mother, highlighting a mutation in the SLC30A2 gene, supports the diagnosis. Once it has been established, the treatment relies on zinc supplementation if the child is exclusively breastfed. Zinc supplementation can later be gradually replaced by the introduction of a diversified diet, which will allow other sources of zinc to meet the child’s metabolic needs.^6^

## Case

**Figure 1.**
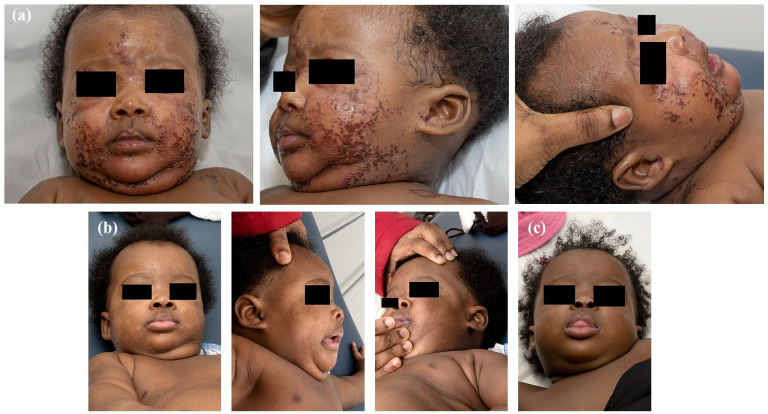
(a) At baseline. (b) After 8 weeks of zinc supplementation. (c) After 6 months of zinc supplementation.

A 3-month-old girl first presented in the emergency department in November 2023 for a skin eruption progressing for a month. She had erythematous and erosive papules and plaques in her face and bilateral elbows, some adopting an annular and reticulated appearance. The lesions had a lacquered aspect with overlying crusts. The right ear and the periocular, perinasal and peribucal regions were affected (Figure 1(a)). The hands, feet, buttocks, genitalia and mucous membranes were spared.

There was no significant personal or familial medical history. The mother was known for a stable immune thrombocytopenic purpura and the father for a treated infection with human immunodeficiency virus. The infant was exclusively breastfed and had normal height, weight and psychomotor development. The lesions were asymptomatic and improved slightly with emollients plus fusidic acid ointment. The child had no other symptoms, including fever, diarrhea or alopecia, and was well appearing.

Blood tests revealed low levels of plasmatic alkaline phosphatase and zinc. A diagnosis of TNZD was made based on clinical and laboratory findings, considering that the child was exclusively breastfed. Zinc gluconate was started to aim for an elemental zinc dose of 1 mg/kg/day. A rapid clinical response was observed in only a few days and a resolution of cutaneous signs in ~8 weeks (Figure 1(b)).

To support the diagnosis, mutation of the SLC30A2 gene in the mother was tested. Unfortunately, a variant of uncertain significance was found and the diagnosis could not be confirmed.

After 6 months of zinc supplementation, no recurrence of skin lesions was observed (Figure 1(c)). Zinc gluconate was stopped since the child had started a diversified diet. The zinc blood level was also normal. Since the mother became pregnant again, a dosage of the breast milk zinc concentration is planned soon after delivery to confirm the diagnosis and to start zinc supplementation in her newborn, if necessary.

## Discussion

TNZD is a rare condition that can mimic clinical manifestations of AE in exclusively breastfed infants. The clinical manifestations and natural history of the disease in our patient are consistent with the literature. Most infants have periorificial and/or acral, shellac-like, eroded papules or plaques.^7^ Other symptoms and signs are possible, such as alopecia, paronychia, cheilitis, stomatitis and diarrhea.^7^ If left untreated, failure to thrive, hypogonadism, neuropsychiatric troubles, difficulty to heal wounds, immune dysregulation and even death may occur.^8^

The diagnosis is made with a typical clinical presentation in an exclusively breastfed child with low plasmatic phosphatase alkaline and zinc levels, combined with a rapid cutaneous response to zinc supplementation. A genetic testing to find the mutation in SLC30A2 in the mother’s plasma or a low zinc concentration in the breast milk supports the diagnosis. However, such testing can represent a challenge. As in our case, it is possible to find some variants of unknown significance since the genetic aspect of this disorder is not yet completely understood.^7^ On the other hand, zinc concentration in breast milk can vary depending on the technique for extraction or analysis.

Treatment of TNZD is limited to the period during which the child is exclusively breastfed. The recommended zinc daily ingestion is around 2 mg for infants.^9^ For TNZD, treatment with 1–2 mg/kg/day of elementary zinc is recommended in children^9^ compared to AE which is 3 mg/kg/day.^4^ Many formulations are possible, such as zinc gluconate (14.3% of elementary zinc), zinc acetate (30% of elementary zinc), zinc oxide (80% of elementary zinc) or zinc sulfate (23% of elementary zinc).^4,10,11^

In conclusion, it is important for clinicians to have a high suspicion index to diagnose and promptly treat this disease to avoid cutaneous and extra-cutaneous complications until the child is weaned from exclusive breastfeeding. Our case supports the classical skin findings of this condition and the necessity to provide follow-up to mothers likely to have other children to allow earlier treatment.
